# Chronic Heat Stress Induces Immune Response, Oxidative Stress Response, and Apoptosis of Finishing Pig Liver: A Proteomic Approach

**DOI:** 10.3390/ijms17050393

**Published:** 2016-05-11

**Authors:** Yanjun Cui, Yue Hao, Jielei Li, Weiguang Bao, Gan Li, Yanli Gao, Xianhong Gu

**Affiliations:** 1 Institute of Animal Sciences, Chinese Academy of Agricultural Sciences, No. 2 Yuanmingyuan West Road, Beijing 100193, China; cuiyanjun196@163.com (Y.C.); haoyueemail@163.com (Y.H.); lijielei123@sina.cn (J.L.); lg0071@126.com (G.L.); gaoyanli.025@163.com (Y.G.); 2 College of Animal Science and Technology, Henan University of Science and Technology, Luoyang 471003, China; baoweiguang2013@163.com

**Keywords:** liver, immune defense, oxidative stress, animal welfare, proteome, pig (*Susscrofa*)

## Abstract

Heat stress (HS) negatively affects human health, animal welfare, and livestock production. We analyzed the hepatic proteomes of finishing pigs subjected to chronic heat stress (HS), thermal neutral (TN), and restricted feed intake conditions, identifying differences between direct and indirect (via reduced feed intake) HS. Twenty-four castrated male pigs were randomly allocated to three treatments for three weeks: (1) thermal neutral (TN) (22 °C) with *ad libitum* feeding; (2) chronic HS (30 °C) with *ad libitum* feeding; and (3) TN, pair-fed to HS intake (PF). Hepatic proteome analysis was conducted using two-dimensional gel electrophoresis and mass spectrometry. Both HS and PF significantly reduced liver weight (*p* < 0.05). Forty-five hepatic proteins were differentially abundant when comparing HS with TN (37), PF with TN (29), and HS with PF (16). These proteins are involved in heat shock response and immune defense, oxidative stress response, cellular apoptosis, metabolism, signal transduction, and cytoskeleton. We also observed increased abundance of proteins and enzymes associated with heat shock response and immune defense, reduced the redox state, enhanced multiple antioxidant abilities, and increased apoptosis in HS liver. Heat-load, independent of reduced feed intake, induced an innate immune response, while food restriction caused stress and cellular apoptosis. Our results provide novel insights into the effects of chronic HS on liver.

## 1. Introduction

Increasing global warming has led to increased research on the detrimental effects of heat stress (HS) on animal welfare and livestock production. Pigs undergo HS as the ambient temperature exceeds their thermal neutral zone (16–22 °C for growing-finishing pigs) [1]. Compared to other animals, pigs are more sensitive to HS due to their high metabolic heat production, quick fat deposition, and lack of sweat glands [2]. HS in pigs decreases food intake, body weight (BW) gain, and meat quality, all of which may account for large economic losses [3,4]. For instance, HS is estimated to cost the US swine industry losses of over $300 million annually [5].

When exposed to a hot environment, diverse physiological mechanisms are adjusted in the thermoregulatory system of animals. Extensive changes at the molecular level also occur during HS. Previous studies have demonstrated that HS results in the increased expression of heat shock proteins (HSPs), oxidative damage, and changes to intracellular signal transduction [6]. Moreover, recent studies indicated that the increased expression of HSPs (HSP70, HSP90, and HSP27) and heat shock transcription factors (HSF1 and HSF2) contribute towards improving the thermal tolerance of pigs [7,8,9]. In addition, TLR2 and TLR4 (TLRs, Toll-like receptors) may mediate immune dysfunction in heat-stressed pigs [10].

Pigs subjected to high ambient temperature challenges tend to reduce nutrition and caloric intake. This response aims to reduce digestion and metabolic heat production [11], but may also affect physiological metabolism. Studies using swine pair-fed models matched to acute HS feed intake levels showed that most changes to the intestinal proteome are mediated by higher core temperatures (direct stressor) rather than reduced feed intake (indirect stressor) [12]. Consequently, both HS and food restriction generate stress conditions that cause the derangement of metabolic and behavioral homeostasis in animals. Thus, it is of interest to understand not only how higher core temperatures alter hepatic function, but also the role of reduced feed intake.

A recent study by our research group [13,14] demonstrated that chronic mild HS (30 °C for three weeks) in finishing pigs leads to decreased feed intake and daily body weight gain, in parallel to increased rectal temperature, respiration rate, and plasma cortisol, as well as decreased plasma free triiodothyronine and growth hormone. These parameters are commonly considered indicators of the consequences of HS on animal physiology [15]. Thus, our results indicated that the finishing pigs entered moderate hyperthermia. Response to HS is a complex biological process that involves multiple proteins and signaling pathways. Therefore, further investigation is required on HS-response mechanisms, particularly at the proteomic and genomic level, to determine the precise response mechanism linked to chronic HS. However, limited studies exist focusing on the molecular mechanisms underlying the effects of chronic HS on the livers of finishing pigs, which are important food animals and biomedical models.

Thus, this study aimed to investigate the proteomic response of liver to chronic HS and to determine whether HS has a direct or indirect effect on hepatic physiological metabolism in finishing pigs (mediated by reduced feed intake). Specifically, we investigated changes to protein expression in liver tissue, because it is critical for digestion, metabolism, immunity, and utilization of feed nutrients and a prime target of tissue injury from environmental challenges, such as HS [16]. Our results are expected to provide new insights into the molecular mechanisms regulating the effects of chronic HS on mammal livers.

## 2. Results

### 2.1. Liver Weight

Absolute liver weight (ALW) was significantly lower (*p* < 0.05) in PF and HS pigs than in thermal neutral (TN) counterparts, whereas ALW was similar when comparing HS with PF (Table 1). The same trend was detected for relative liver weight (RLW, absolute liver weight (kg) corrected for body weight (kg)) as ALW in all three groups.

### 2.2. Proteomic Changes to the Liver in Response to Heat Stress (HS)

**Figure 1:**
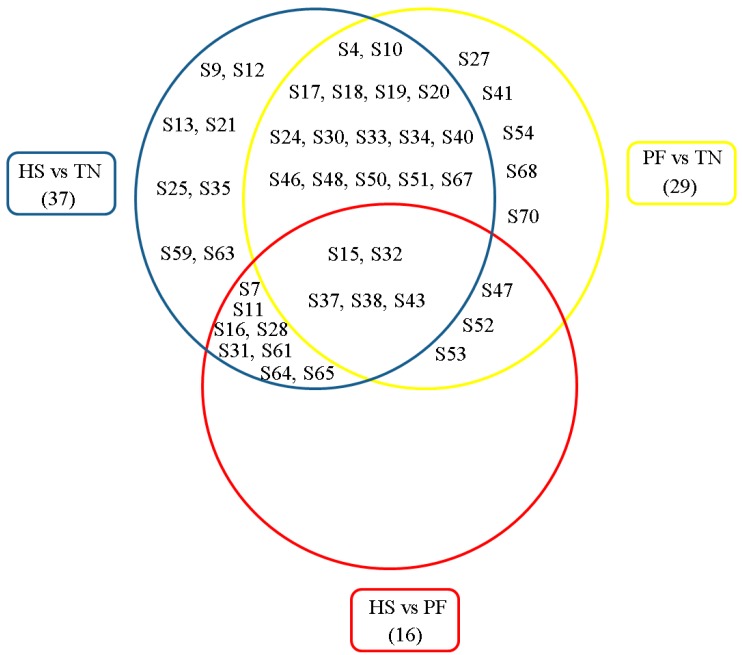
Protein information from Table 2 is presented using a Venn diagram analysis. Thirty-seven, 29, and 16 protein spots changed when comparing HS with TN (37), PF with TN (29), and heat stress (HS) with PF (16), respectively. TN = thermal neutral; HS = heat stress; PF = Pair-fed; S = spot. The spot numbers of the identified proteins are the same as those described in Table 2.

**Figure 2:**
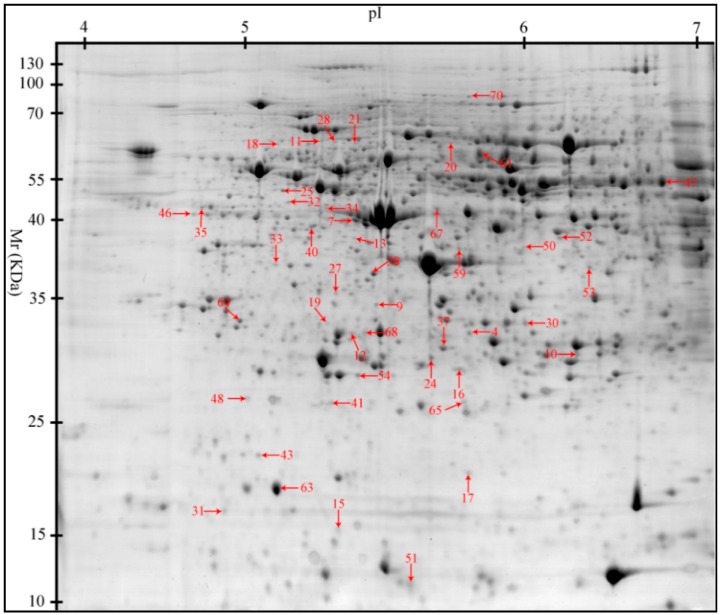
Protein profile patterns in the liver of finishing pigs subjected to chronic heat stress or reduced feed intake. Protein spots showing significant differences (1.3-fold, *p* < 0.05) were cut out and identified by Liquid chromatography-mass spectroscopy (LC–MS/MS). Protein spots of differential abundance with known identities are marked with red arrows.

**Figure 3:**
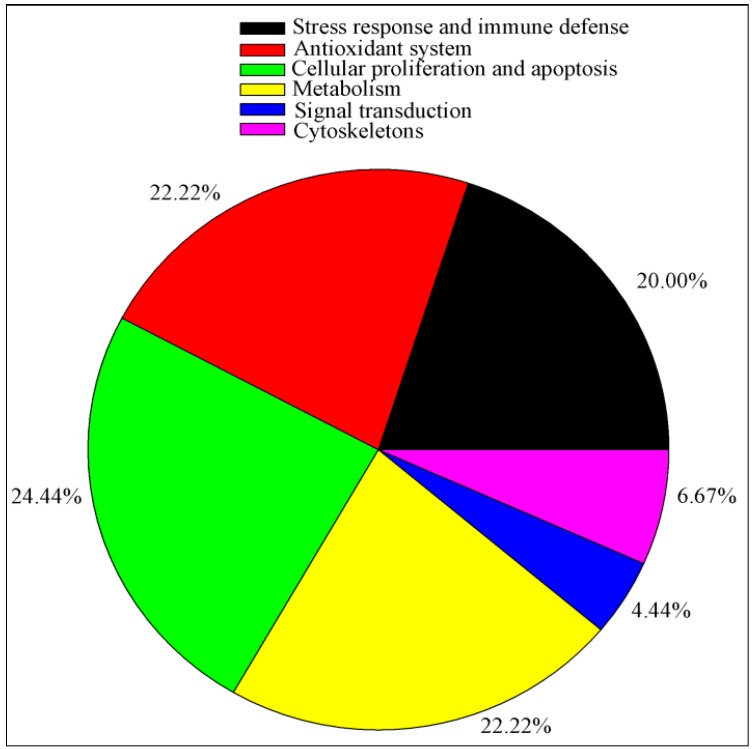
Functional classification of the differentially expressed proteins identified from the liver of finishing pigs subjected to chronic heat stress or reduced feed intake. The color codes represent different protein functional groups.

A 2-D approach was used to detect changes to the protein profiles of the three treatments. A total of 1489 protein spots were detected per 2-D gel. Of these protein spots, 45 were differentially expressed (≥1.3-fold change, *p* < 0.05) among the three treatments. Thirty-seven, 29, and 16 protein spots changed when comparing HS with TN (37), PF with TN (29), and HS with PF (16), respectively (Figure 1). Differentially expressed proteins were identified using Liquid chromatography-mass spectroscopy (LC–MS/MS). Table 2 presents the biochemical information about these identified protein spots, while their appearance on the gel images is shown in Figure 2. These proteins were classified into six groups based on their biological functions: (1) stress response and immune defense (20.00%); (2) antioxidant system (22.22%); (3) cellular proliferation and apoptosis (24.44%); (4) metabolism (22.22%); (5) signal transduction (4.44%); and (6) cytoskeleton (6.67%) (Figure 3). The functions related to the stress response and immune defense, antioxidant system, and cellular apoptosis were predominant, representing 67% of the differential proteins.

#### 2.2.1. Stress Response and Immune Defense

**Figure 4:**
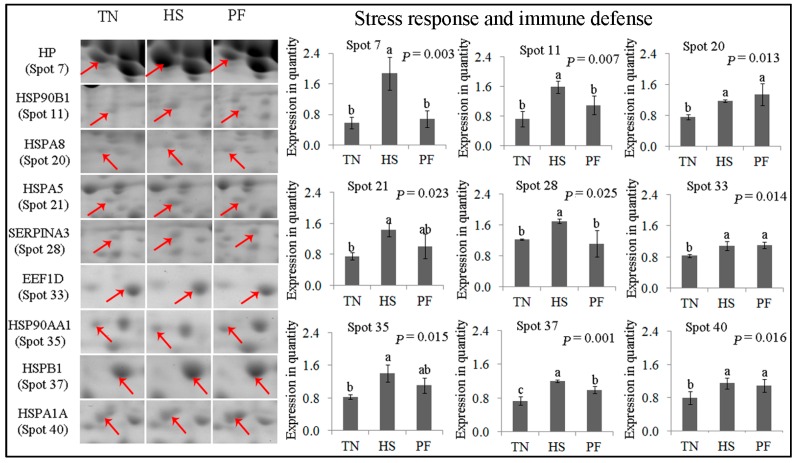
Quantification of differentially expressed proteins associated with stress response and immune defense. Data are mean ± SD; *n* = 8 for each group. Within a panel, a, b, c means without a common letter differ (*p* < 0.05). The spot numbers of the identified proteins are the same as those described in Table 2. These altered spots are marked by red arrows. For an explanation of abbreviations, please refer to Table 2. TN = thermal neutral; HS = heat stress; PF = Pair-fed.

Nine proteins spots were related to the stress response and defense system (Figure 4). These spots were HP (spot 7). HSP90B1 (spot 11), HSPA8 (spot 20), HSPA5 (spot 21), SERPINA3 (spot 28), EEF1D (spot 33), HSP90AA1 (spot 35), HSPB1 (spot 37), and HSPA1A (spot 40). Of these spots, the abundance of HSP90B1 (spot 11), HP and SERPINA3 (spot 28) was higher (*p* < 0.05) in HS pigs compared to TN and PF pigs, while no difference was detected between TN and PF pigs. Compared with TN, both HS and food restriction induced the up-regulation of EEF1D (spot 33), HSPA8 (spot 20) and HSPA1A (spot 40) (*p* < 0.05); however, no difference was detected between HS and PF. HSPB1 (spot 37) expression was higher in PF pigs compared to TN pigs, but was lower in PF pigs compared to HS pigs (*p* = 0.001). HSP90AA1 (spot 35) and HSPA5 (spot 21) were more up-regulated in HS compared to TN (*p* < 0.05), whereas no difference was detected for TN *vs.* PF and HS *vs.* PF.

#### 2.2.2. Antioxidant System

**Figure 5:**
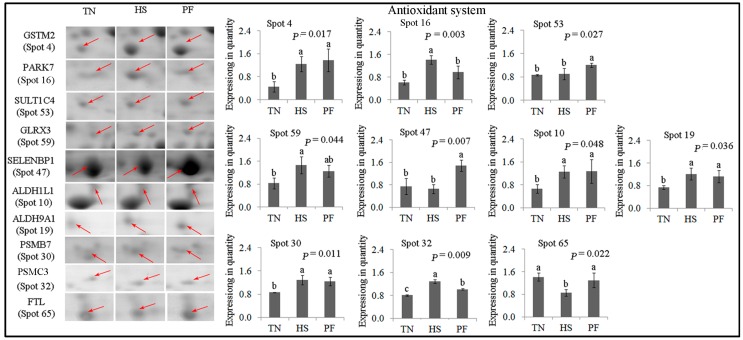
Quantification of differentially expressed proteins associated with antioxidant system. Data are mean± SD; *n* = 8 for each group. Within a panel, a, b, c means without a common letter differ (*p* < 0.05). The spot numbers of the identified proteins are the same as those described in Table 2. These altered spots are marked by red arrows. For an explanation of abbreviations, please refer to Table 2. TN = thermal neutral; HS = heat stress; PF = Pair-fed.

Ten protein spots are critical in the antioxidant pathway (Figure 5). FTL (spot 65) levels were lower in HS pigs compared to the other two treatments (*p* = 0.022), while PARK7 (spot 16) levels were higher in HS pigs compared to the other two treatments (*p* = 0.003). GLRX3 (spot 59) was up-regulated in HS compared to TN, but not in TN *vs.* PF or HS *vs.* PF (*p* < 0.05). GSTM2 (spot 4), ALDH1L1 (spot 10) and ALDH9A1 (spot 19) were up-regulated in the HS and PF groups compared to TN (*p* < 0.05), while no difference was detected between HS and PF. SELENBP1 (spot 47) and SULT1C4 (spot 53) were up-regulated in the PF group compared with TN and HS (*p* < 0.05). PSMB7 (spot 30) was up-regulated in HS and PF, while no difference was detected when comparing HS with PF (*p* < 0.05). PSMC3 (spot 32) expression was higher in HS than PF and TN, with food restriction also increasing the expression of this spot (*p* < 0.01).

#### 2.2.3. Cellular Proliferation and Apoptosis

**Figure 6:**
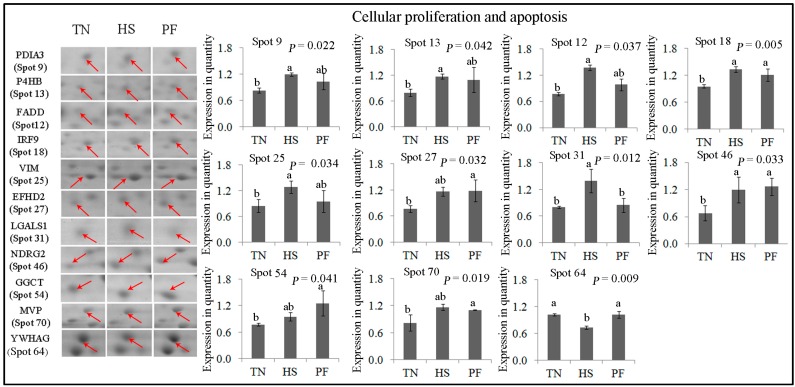
Quantification of differentially expressed proteins associated with cellular proliferation and apoptosis. Data are mean ± SD; n = 8 for each group. Within a panel, a, b, c means without a common letter differ (*p* < 0.05). The spot numbers of the identified proteins are the same as those described in Table 2. These altered spots are marked by red arrows. For an explanation of abbreviations, please refer to Table 2. TN = thermal neutral; HS = heat stress; PF = Pair-fed.

Eleven protein spots related to cellular proliferation and apoptosis were affected by the PF and HS treatments (Figure 6). FADD (spot 12), VIM (spot 25), P4HB (spot 13), and PDIA3 (spot 9) expression was up-regulated in HS compared to TN (*p* < 0.05), whereas PF protein expression remained unchanged. NDRG2 (spot 46) and IRF9 (spot 18) expression was elevated in PF and HS compared with TN (*p* < 0.05), but no difference was observed between PF and HS. LGALS1 (spot 31) expression was higher in HS compared to the other 2 groups (*p* = 0.012), whereas YWHAG (spot 64) expression was lower in HS compared to the other 2 groups (*p* = 0.009). No difference was detected between TN and PF for these 2 proteins. EFHD (spot 27), GGCT (spot 54) and MVP (spot 70) expression remained unchanged following HS, but their expressions were up-regulated by PF (*p* < 0.05).

#### 2.2.4. Metabolism

**Figure 7:**
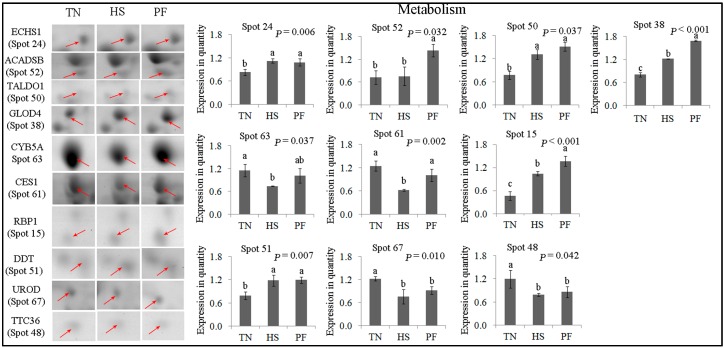
Quantification of differentially expressed proteins associated with metabolism. Data are mean ± SD; *n* = 8 for each group. Within a panel, a, b, c means without a common letter differ (*p* < 0.05). The spot numbers of the identified proteins are the same as those described in Table 2. These altered spots are marked by red arrows. For an explanation of abbreviations, please refer to Table 2. TN = thermal neutral; HS = heat stress; PF = Pair-fed.

Ten protein spots were related to metabolism, including ECHS1 (spot 24), TALDO1 (spot 50), ACADSB (spot 52), GLOD4 (spot 38), CYB5A (spot 63), CES1 (spot 61), UROD (spot 67), TTC36 (spot 48), RBP1 (spot 15), and DDT (spot 51) (Figure 7). ECHS1 (spot 24) and TALDO1 (spot 50) expression increased following HS and PF compared to TN (*p* < 0.05), but no difference was detected between HS and PF. ACADSB (spot 52) expression was significantly induced by PF (*p* = 0.032), but was not altered by HS. GLOD4 (spot 38) and RBP1 (spot 15) expression was higher in HS group compared to TN, but was lower in HS compared to PF (*p* < 0.01). CYB5A (spot 63) expression was lower in HS compared to TN (*p* = 0.037), but no difference was detected for TN *vs.* PF and HS *vs.* PF. CES1 (spot 61) levels were higher (*p* = 0.002) in HS compared to TN and PF, while no difference was detected between TN and PF.

#### 2.2.5. Signal Transduction

**Figure 8:**
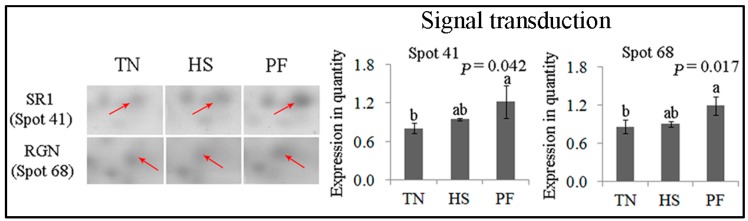
Quantification of differentially expressed proteins associated with signal transduction. Data are mean ± SD; *n* = 8 for each group. Within a panel, a, b, c means without a common letter differ (*p* < 0.05). The spot numbers of the identified proteins are the same as those described in Table 2. These altered spots are marked by red arrows. For an explanation of abbreviations, please refer to Table 2. TN = thermal neutral; HS = heat stress; PF = Pair-fed.

SRI (spot 41) and RGN (spot 68) were involved in signal transduction (Figure 8). The expression of both proteins was significantly increased by PF compared to TN (*p* < 0.05), with the expression remaining unchanged in HS.

#### 2.2.6. Cytoskeleton

**Figure 9:**
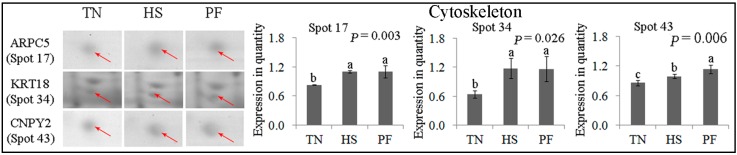
Quantification of differentially expressed proteins associated with cytoskeleton. Data are mean ± SD; *n* = 8 for each group. Within a panel, a, b, c means without a common letter differ (*p* < 0.05). The spot numbers of the identified proteins are the same as those described in Table 2. These altered spots are marked by red arrows. For an explanation of abbreviations, please refer to Table 2. TN = thermal neutral; HS = heat stress; PF = Pair-fed.

Three differentially expressed protein spots were associated with cytoskeleton, including ARPC5 (spot 17), KRT18 (spot 34) and CNPY2 (spot 43) (Figure 9). ARPC5 (spot 17) and KRT18 (spot 34) were up-regulated in HS and PF (*p* < 0.05), with no difference being detected between HS and PF. CNPY2 (spot 43) expression was higher in HS compared to TN, but was lower in HS compared to PF (*p* = 0.006).

### 2.3. Confirmation of Differentially Abundant Proteins by Western Blot

**Figure 10:**
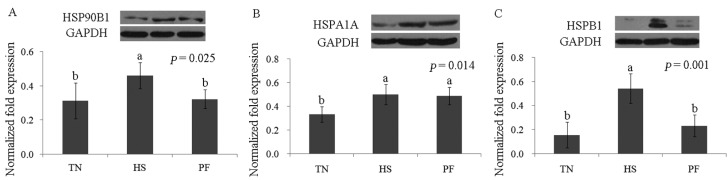
Western blotting analysis of liver proteins, HSP90B1 (**A**), HSPA1A (**B**), and HSPB1 (**C**). Data are mean ± SD, *n* = 8 pigs for each group. Within a panel, a, b, c means without a common letter differ (*p* < 0.05). For an explanation of abbreviations, please refer to Table 2. TN = thermal neutral; HS = heat stress; PF = Pair-fed.

Immunoblotting was performed to verify the proteomics results. The presence of the three stress-response marker proteins (HSP90B1, HSPA1A, and HSPB1) was confirmed using antibodies. The immunoblotting analysis results were generally consistent with the 2-DE results (Figure 10).

### 2.4. Changes to the Redox State and Related Enzymes

**Figure 11:**
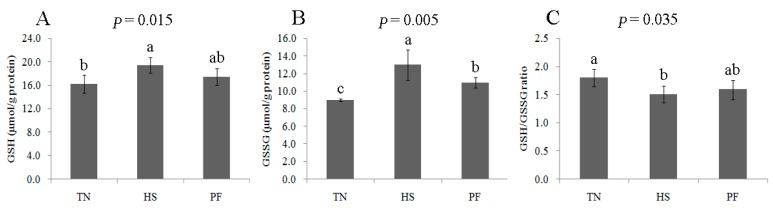
Effects of *ad-libitum* feed intake in thermal neutral conditions (TN; 22 °C), *ad-libitum* feed intake in chronic heat stress conditions (HS; 30 °C), or pair-feeding in thermal neutral conditions (PF) on levels of GSH (**A**), GSSG (**B**) and GSH/GSSG (**C**) in the liver of finishing pigs. Data are mean ± SD; *n* = 8 for each group. Within a panel, a, b, c means without a common letter differ (*p* < 0.05). For an explanation of abbreviations, please refer to Table 2. TN = thermal neutral; HS = heat stress; PF = Pair-fed.

**Figure 12:**
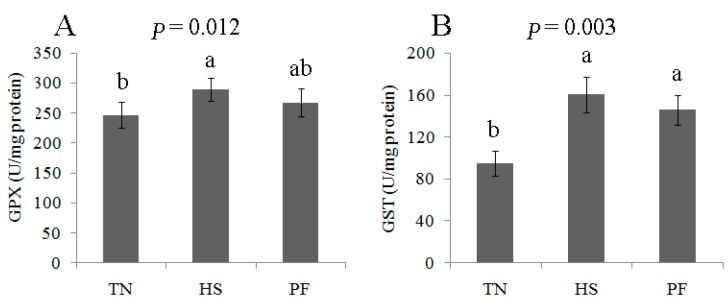
Effects of *ad-libitum* feed intake in thermal neutral conditions (TN; 22 °C), *ad-libitum* feed intake in chronic heat stress conditions (HS; 30 °C), or pair-feeding in thermal neutral conditions (PF) on GPX (**A**) and GST (**B**) activities in the liver of finishing pigs. Data are mean± SD; *n* = 8 for each group. Within a panel, a, b means without a common letter differ (*p* < 0.05). For an explanation of abbreviations, please refer to Table 2. TN = thermal neutral; HS = heat stress; PF = Pair-fed.

The proteomics analysis indicated the occurrence of oxidative stress induced by HS and PF. This analysis was verified by determining the activities of oxidative stress-related proteins (GST and GPX) and the concentration of reduced glutathione (GSH) and oxidized glutathione (GSSG) in the liver tissue. Compared to TN pigs, the concentrations of GSH and GSSG were elevated by 21 days HS, whereas the GSH/GSSG ratio declined (*p* < 0.05; Figure 11). GST and GPX enzymatic activity was higher in HS pig liver compared to TN pig liver. All parameters were similar when comparing TN with PF and HS with PF (Figure 12).

### 2.5. Bioinformatics Analysis of Differentially Expressed Proteins

**Figure 13:**
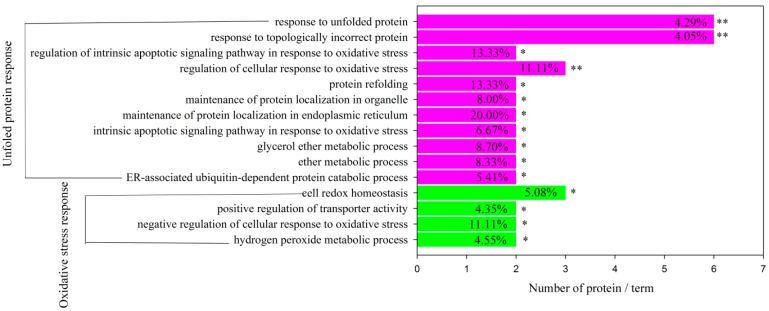
Functional enrichment analysis of the proteins of differential abundance from the liver paper of finishing pigs using the ClueGO software. * *p* < 0.05 and ** *p* < 0.01. For an explanation of abbreviations, please refer to Table 2. TN = thermal neutral; HS = heat stress; PF = Pair-fed.

Gene Ontology (GO) enrichment analysis and functional annotation currently are useful techniques for analyzing large proteomic and genomic datasets. Significantly overrepresented GO terms determine the putative biological events of data, providing a preliminary overview of the liver proteome. Functional enrichment analysis of all differential proteins was conducted using the ClueGo software. Two major functional groups were significantly enriched, including the oxidative stress response and unfolded protein response (UPR) (Figure 13). PARK7 (spot 16), GSTM2 (spot 4), GLRX3 (spot 59), HSPB1 (spot 37), HP (spot 7), P4HB (spot 13), and PDIA3 (spot 9) were significantly enriched in the oxidative stress response. HSP90B1 (spot 11), HSPA5 (spot 21), HSPB1 (spot 37), HSP90AA1 (spot 35), HSPA1A (spot 40), P4HB (spot 13), PDIA3 (spot 9), and HSPA8 (spot 20) were significantly enriched in the unfolded protein response (UPR).

The Kyoto Encyclopedia of Genes and Genomes (KEGG) pathway enrichment analysis of the differentially expressed proteins reveals protein functional information in the metabolic pathway. KEGG pathway analysis showed that 11 differential proteins were significantly enriched within the five pathways, which contribute to multiple biological processes associated with ascorbate and aldarate metabolism, fatty acid degradation, proteasome, unfolded protein response and antigen processing and presentation (Table 3).

**Figure 14:**
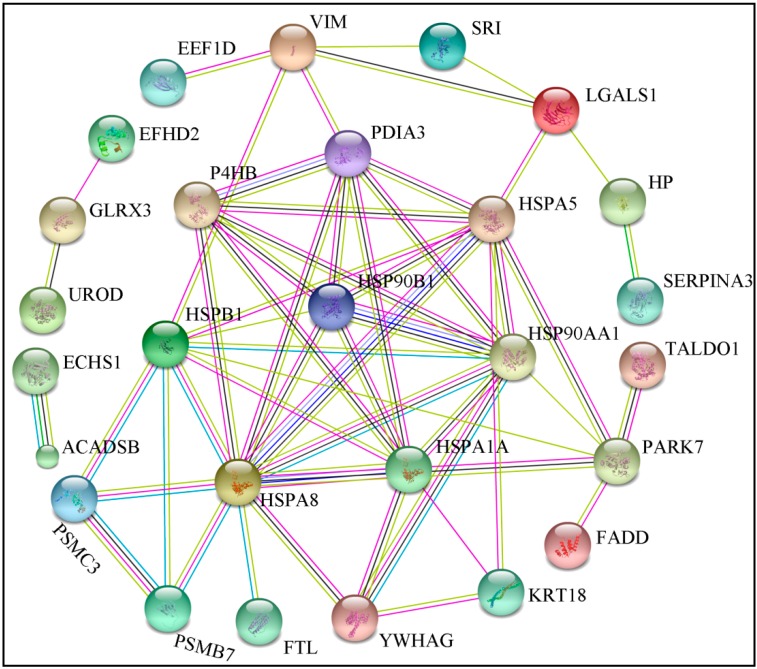
Biological interaction network of the identified differentially expressed proteins from the liver of finishing pigs. A red line indicates fusion evidence; a green line—neighborhood evidence; a blue line—co-occurrence evidence; a purple line—experimental evidence; a yellow line—text mining evidence; a light blue line—database evidence; and a black line—coexpression evidence.

Proteins function as elementary parts of protein complexes in living cells; however, proteins do not act independently. Twenty-seven proteins were recognized as key nodes that have various relationships in protein–protein interactions (PPI) (Figure 14). Using the online tools of STRING 9.1, we demonstrated that nine proteins were related to the stress response and immune defense; specifically, HSPA5 (spot 21), HSP90A1A (spot 35), HSPB1 (spot 37), HSPA1A (spot 40), HSPA8 (spot 20), HSP90B1 (spot 11), EEF1D (spot 33), HP (spot 7), and SERPINA3 (spot 28). The second most represented group included proteins related to cellular apoptosis; specifically, FADD (spot 12), PDIA3 (spot 9), P4HB (spot 13), VIM (spot 25), LGALS1 (spot 31), EFHD2 (spot 27), and YWHAG (spot 64). The third most represented group included proteins involved in metabolism; specifically, ACADSB (spot 52), ECHS1 (spot 24), TALDO1 (spot 50), and UROD (spot 67), with each protein being linked to the network. Five proteins were involved in antioxidant systems; FTL (spot 65), PARK7 (spot 16), PSMB7 (spot 30), PSMBC3 (spot 32), and GLRX3 (spot 59). KRT18 (spot 34) were related to cytoskeletons; SRI (spot 41) was involved in signal transduction.

For an explanation of abbreviations, please refer to Table 2.

## 3. Discussion

Our results showed a significant decline in the weight of finishing pig livers exposed to chronic HS (30 °C) and food restriction for three weeks, demonstrating that the animals were subjected to stress conditions that retarded liver growth. Chronic HS and PF caused a change in the expression of hepatic proteins that are mainly involved in the heat shock response, immune defense, oxidative stress response, and cellular apoptosis. To our knowledge, this study is the first to demonstrate how chronic-HS-induced alters the hepatic proteome of finishing pigs.

HSPs and acute phase proteins (APPs) are involved in the stress response and immune defense. HSPs are a family of proteins that restore protein homeostasis and contribute to cell survival. Thus, these proteins have various roles, including chaperoning, protein folding and transport, inhibiting cellular apoptosis, and protecting cells against thermal or oxidative stress [17]. Our results support their involvement in these roles by showing that several HSPs are upregulated due to HS, all of which are heat-inducible (namely, HSP90B1, HSP90AA1, HSPA8, HSPA1A, HSPB1 (GRP94), and HSPA5 (GRP78)). Exposure to chronic HS is associated with an increase in HSP synthesis, which protects cells against hyperthermia [18].

Food restriction also induced increases in HSPA8 and HSPA1A gene expression. Our results support those of Aly *et al.* [19], who demonstrated that HSP expression increased in animal tissue maintained under caloric restriction. Low caloric intake is considered a mildly stressful condition that induces a survival response, enhancing the defense mechanisms of organisms [20].

A novel and important finding of this study was the up-regulation of HSP90B1 (known as GRP94/gp96), HP, and SERPINA3 for HS *vs.* PF and HS *vs.* TN, but not for TN *vs.* PF. Therefore, HS alters the expression level of these proteins independently of PF. GRP94 is the most abundant glycoprotein in the endoplasmic reticulum (ER). This protein is involved in the processing and transport of secreted proteins and is required for the proper folding of toll-like receptors (TLRs). This protein is critical for the initiation of the innate immune response [21]. Thus, it may be associated with the innate immune response induced by HS. This hypothesis requires confirmation; however, the results of the present study provide preliminary support. For instance, HP and SERPINA3, which belong to APPs, are also glycoproteins that are mainly synthesized by hepatocytes following stimulation by pro-inflammatory cytokines, including interleukin-1β (IL-1β) and IL-6 [22]. These cytokines are produced by liver-resident macrophages activated by TLRs [23]. Furthermore, TLRs mediate innate immune recognition and trigger the immune response. Therefore, we hypothesize that HS induces the inflammatory response, releasing pro-inflammatory cytokines modulated by TLRs, which stimulate hepatocytes to produce innate immune proteins, such as HP and SERPINA3, for protection. The up-regulation of GRP94 may help maintain the proper folding of TLRs during HS. Several studies support this hypothesis, showing that the TLR signaling pathway is activated under HS in mammals, including pigs [24,25].

The oxidative stress/antioxidant pathway may also be influenced by HS. In our study, HS changed the expression of anti-oxidative proteins and reduced the redox state, with this interpretation being based on an increase in the GSH: GSSG ratio in the livers of the finishing pigs. The GSH/GSSG ratio is an indicator of oxidative stress [26]. This ratio was lower in the liver of HS pigs compared to PF and TN pigs, with this result being consistent with that reported for chronic HS mouse liver [27]. Thus, chronic heat exposure increases oxidative stress. Our results supported this hypothesis based on our findings of increased GST and GPX activity in the liver following chronic HS (Figure 12). The increase in both GST and GPX activity may be an adaptive mechanism underlying the increase in oxidative stress [28].

**Figure 15:**
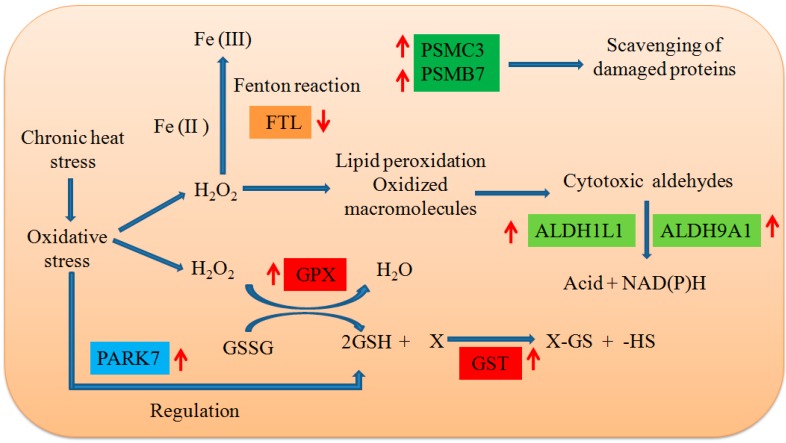
Differentially expressed proteins involved in anti-oxidant adaptive mechanism. The red arrows indicate up- or down-regulated proteins in response to the chronic heat stress. Protein names for the symbols used are defined in Table 2. The same background color behind protein names means the similar biological function of proteins.

Our proteomic data further confirmed that chronic HS induces oxidative stress, activating the anti-oxidant adaptive mechanism (Figure 15). The expression levels of eight identified proteins [including FTL (ferritin) GST, ALDH1L1, ALDH9A1, GLRX3, PARK7 (DJ-1), PSMC3 and PSMB7] were correlated with the oxidative stress response.

Ferritin primarily keeps iron in a soluble and non-toxic form, and transports it to areas of the body where it is required [29]. Free iron, via the Fenton reaction, is involved in the formation of the highly damaging ROS hydroxyl radical [30]. The downregulation of ferritin in the liver may increase free iron levels, leading to increased oxidative stress.

GST is crucial in the glutathione (GSH) redox cycle, and catalyzes the conjugation of glutathione to a variety of electrophiles (such as reactive oxygen species) to protect biomacromolecules (including proteins, especially those containing SH-containing cysteine) against oxidation [31]. Thus, when oxidative stress levels are high, proteins are oxidized [32], and GST expression is usually induced [33]. Glutaredoxin3 (GLRX3) is a glutathione (GSH)-dependent reductase of the disulfide, containing two [2Fe–2S]^2+^ clusters. This special structure allows it to detect redox during signal transduction in response to stress signals by reactive oxygen and nitrogen species [34]. PARK7 (DJ-1) functions as a redox-dependent protein chaperone to alleviate molecular insults induced by ROS bursts through the up-regulation of GSH synthesis [35], as observed in the current study. Collectively, the elevated expression of the three proteins (GST, GLRX3 and PARK7) related to glutathione metabolism may be an adaptive mechanism secondary to the increase of oxidative stress induced by chronic HS in pig liver.

Prolonged exposure to high temperatures promotes protein damage for cells [36]. This impedes cellular function, leading to the apoptosis of cells [37]. Therefore, these damaged proteins must be broken down for cell survival. In the current study, PSMC3 and PSMB7 were up-regulated in chronic HS. Both proteins are subunits of the 26S proteasome, which is involved in the direct degradation of oxidized or misfolded proteins or via the ubiquitin-proteasomal pathway [38]. The elevated expression of the both proteins because of chronic HS might facilitate the removal of damaged proteins, providing an efficient way for cells to survive chronic HS conditions. This result is similar to that reported for the up-regulation of PSMB5, which is also a subunit of the 26S proteasome, under chronic HS of the mouse liver [27].

Aldehyde dehydrogenases (ALDHs) are a group of NAD(P)^+^-dependent enzymes that oxidize aliphatic and aromatic aldehydes to their corresponding carboxylic acids via a pyridine nucleotide-dependent reaction. ALDHs act as “aldehydes scavengers,” enabling them to protect cells against oxidative stress, particularly stress caused by aldehydes [39]. Thus, the enhanced expression of ALDH1L1 and ALDH9A1, which are isozymes of aldehyde dehydrogenases (ALDHs), may combat oxidative stress induced by chronic HS in pig liver.

Homeostatic balance between cell proliferation and apoptosis is essential for liver regeneration. An important finding of this study was that HS affects the expression of apoptosis-related proteins, including P4HB (PDIA1), PDIA3, YWHAG, IRF9, VIM, LGALS1, and NDGR2. These proteins were the most represented out of all of the identified differentially expressed proteins. Chronic HS induces (endoplasmic reticulum) ER stress, as indicated by an increase in the expression of the ER-stress marker proteins, GRP78 and GRP94 [40] by the present study. ER stress responses commonly lead to an unfolded protein response (UPR), which induces the up-regulation of P4HB and PDIA3 [41]. A recent study has demonstrated that persistent ER stress causes the release of P4HB from the ER to mitochondria, leading to the initiation of apoptosis [42]. Therefore, chronic HS in the present study may induce the accumulation of P4HB above threshold levels, triggering apoptosis.

Fas-associated death domain protein (FADD) has a regulatory role in liver cellular apoptosis. At the beginning of apoptosis, the interaction of FADD and death domain-containing receptors (such as Fas, TNF-R1, DR3, DR4, and DR5) initiate apoptosis [43]. The FADD dominant negative mutant also inhibits TNFR-1induced cell death [44]. The overexpression of FADD indicates that HS is responsible for apoptosis mediated by FADD. Vimentin (VIM) is a major intermediate filament (IF) protein of the mesenchymal cells. Under quiescent conditions, polymeric vimentin maintains cellular integrity. However, under stress and stimulatory conditions, vimentin phosphorylation impairs the steady state of vimentin in favor of increased free depolymerized vimentin. Furthermore, vimentin cleavage releases potential proapoptotic proteolytic fragments that markedly enhance apoptosis [45]. In the current study, the high level of soluble vimentin (free depolymerized vimentin) in heat-stressed liver indicates increased degradation, facilitating apoptosis. The 14-3-3 proteins are a group of dimeric acidic proteins that are relatively conserved in all eukaryotes. Previous studies have demonstrated that the 14-3-3 epsilon protein (YWHAG) is one of the caspase-3 substrates. The cleavage of the 14-3-3 protein promotes cell death by releasing the associated BAD from the 14-3-3 protein, facilitating BAD translocation to the mitochondria and its interaction with Bcl-x [46]. Based on our study results, we hypothesize that the down-regulation of 14-3-3 proteins leads to the cytoplasmic release of phosphorylated BAD protein, inducing apoptosis. Galectin-1 (LGALS1) also induces the apoptosis of activated T cells, suggesting a novel role for galectin-1 in the modulation of the immune response. Given its overexpression in HS compared to both PF and TN (but not when comparing PF with TN) indicates that HS (independent of restricted food intake) triggers the immune response regulated by galectin-1-induced apoptosis.

Of interest, this study detected the up-regulation of IRF9 and NDRG2 in PF. These two proteins are closely associated with apoptosis. IRF9, which is also called interferon (IFN) regulatory factor 9, is the key transcriptional factor used to elicit the antiproliferative activity of IFN-α in the JAK-STAT pathway [47]. IRF9 overexpression facilitates IFN-α-induced apoptosis in T98G cells.

The N-myc downstream-regulated gene 2 (NDRG2) is the second member of the NDRG family of genes that is involved in cell differentiation, proliferation, death, and migration [48,49]. NDRG2 gene expression regulates the stress response [50]. Previous studies have demonstrated that NDRG2 is involved in hypoxia-inducible factor 1 (HIF-1)-mediated apoptosis, which silences or enforces the expression of NDRG2, and could strongly inhibit or enhance apoptosis under hypoxia [50,51]. Food restricted pigs may be subject to some level of hypoxia and stress [52]. The up-regulation of NDRG2 in our study indicates that chronic feed restriction triggers hypoxia-inducible factor 1 (HIF-1)-mediated apoptosis. A previous study also suggested that restricted food intake induces apoptosis in animal liver [53].

In living cells, proteins build complex networks to fulfill different functions through protein–protein interactions, modifications, and protein regulation [54]. The biological interaction network (BIN) clearly demonstrates that proteins related to the stress response, immune defense, oxidative stress response, and cellular apoptosis account for approximately 67% of the BIN, demonstrating that the liver is involved in a range of molecular adaptive responses during HS. These results support the results of GO functional enrichment analysis.

## 4. Materials and Methods

### 4.1. Animals and Experimental Design

Twenty-four castrated male DLY pigs (crossbreeds of Landrace × Yorkshire sows and Duroc boars) were randomly selected from 8 litters at a pig breeding farm (Beijing, China), and were transported to the State Key Laboratory of Animal Nutrition (Beijing, P. R. China). Individual pig body weights were 79.00 ± 1.50 kg. Pigs were randomly allocated to 1 of 3 treatments (8 pigs per treatment): (1) thermal neutral (TN) (22 °C) with *ad libitum* feeding; (2) chronic HS (HS) (30 °C) with *ad libitum* feeding); and (3) TN but pair-fed (PF) to reflect the nutrient intake of the HS pigs. The extent of food restriction (pair-fed) corresponded to 80% of the dietary intake by control pigs, which is a normal response of pigs exposed to HS. Four pigs were housed in an artificial climate chamber (2.1 × 4.8 m^2^, luminance 100 lx, photoperiod 14 h light, humidity 55% ± 5%) and 6 artificial climate chambers were used. All animals were fed with standard feed according to the NRC (1998) recommendations. The feed contained no antibiotics (Table 4).

Before the experiment, the animals were allowed to acclimate to 22 °C in the artificial chamber for 7 days. To minimize damage caused by ambient heat loads (30 °C) in the HS group, the artificial temperature climate of the chamber was gradually increased to and kept at 27 °C on day 1, and then raised to 28 °C on day 2. Thereafter, the temperature was kept constant at 30 °C, while both TN and PF groups were maintained at 22 °C, until the end of the experiment. The experimental period lasted for 3 weeks. The study was conducted at the State Key Laboratory of Animal Nutrition.

The experiment was performed in accordance with guideline of the Beijing Animal Ethics Committee and received prior approval from the Chinese Academy of Agricultural Sciences Animal Care and Use Committee.

### 4.2. Biological Samples

The pigs were slaughtered by a head-only electric stun tong apparatus (Xingye Butchery Machinery Co. Ltd., Changde, China), and were killed by manual exsanguination. Immediately after slaughter, the livers were removed from all pigs and weighed. Subsequently, the tissues were washed with PBS to remove any blood and contaminants on the surface of the organ. Then, we placed the livers into sterile tubes and we snap froze the tubes in liquid nitrogen. Once in the laboratory, the tubes were stored at −80 °C until biochemical and molecular analyses.

### 4.3. Proteomic Analysis

#### 4.3.1. Liver Protein Extraction

Protein extraction was conducted as described with slight modifications [55]. In brief, frozen samples of liver from all pigs in three groups were crushed in a mortar containing liquid nitrogen. The powder (approximately 100 mg per sample) was transferred to sterile tubes with 1mL lysis buffer (LB; containing 7 M urea, 2 M thiourea, 4% *w*/*v* CHAPS, 1% *w*/*v* DTT, 1% *v*/*v* IPG Buffer pH 4–7, 1% *v*/*v* proteinase inhibitor cocktail). The mixture was sonicated in an ice bath using a Model VCX 500 Ultrasonicater (Sonics & Materials, Newtown, CT, USA) at 20% power output for 10 min with 2-s on and 4-s off cycles. Subsequently, the lysed cell suspension was incubated at room temperature for 1 h to solubilize proteins. After centrifugation at 40,000× g and 4 °C for 40 min, the supernatant protein was collected and its protein concentration was determined according to a modified Bradford assay [56]. The protein concentration was 8.69 ± 0.94 mg/mL.

#### 4.3.2. Two-Dimensional Gel Electrophoresis

A 1 mg protein sample was loaded on a 24 cm IPG strip (immobilized pH gradient, pH 4–7, linear, GE Healthcare) (Amersham Bioscience, Uppsala, Sweden). Each protein sample was assessed in triplicate. Isoelectric focusing (IEF) was carried out at 20 °C for 14 h at 30 V, 2 h at 200 V, 0.5 h at 500 V, 1 h at 1000 V, 3 h at 8000 V, and then held at 8000 V until a total of at least 60,000 V was reached (EttanIPGphorII, GE Healthcare). Focused IPG strips were equilibrated for 15 min in equilibration buffer (6 M urea, 30% glycerol, 2% SDS, 50 mM Tris pH 8.8, 1% DTT) under gentle agitation, and then for an additional 15 min in the same buffer, except that DTT was substituted with 2.5% iodoacetamide. After equilibration, the strips were transferred to vertical slab gels (12% SDS-PAGE) for second-dimensional electrophoresis with the Ettan DALT six gel system (GE Healthcare).

#### 4.3.3. Image Acquisition and Analysis

Gels were fixed for about 8 h in solution (10% (*v*/*v*) acetic acid, 40% (*v*/*v*) ethanol, and 50% (*v*/*v*) water), washed 3 times in water, and then stained with Coomassie colloidal blue G-250 according to the GE handbook (GE Healthcare) with minor modifications. Gel images were acquired with the PowerLook 2100XL color scanner (UMAX Technologies, Atlanta, CA, USA) at a resolution of 16 bits and 300 dpi and were assayed by Image master 2D Platinum Software Version 6.0 (GE Healthcare). To compare the spot quantities between gels accurately, each spot volume was normalized as a percentage of the total volume of all of the spots in the gel. All automatic spot detections in each gel were manually inspected and edited as necessary to confirm the absence of mismatched and unmatched spots. Differentially expressed protein spots were changed abundance by at least ±1.2-fold, with an error probability of *p* < 0.05.

#### 4.3.4. Trypsin Digestion of Protein and Identification of Differentially Expressed Proteins by MS

The protein spots were excised from the gels and destained for 30 min using 100 µL of acetonitrile (50%) and 25 mM NH_4_HCO_3_ (pH 8, 50%) until the gel particles were transparent. The gel particles were dehydrated for 10 min with acetonitrile (100%) and dried for 30 min using a Speed-Vac system (South San Francisco, CA, USA). To prepare the trypsin solution, 2.5 mL of 25 mM NH_4_HCO_3_ was added to 25 µg of trypsin (final concentration of 10 ng/µL, Roche, South San Francisco, CA, USA). The trypsin solution (10 µL) was pipetted onto each dried protein spot, and the dishes were incubated for 1 h at 4 °C. To avoid trypsinauto-digestion, the excess trypsin was removed. Then, the sample was incubated for 12 h at 37 °C. To extract the peptide fragments from the tryptic digests, 30 µL of 5% (*v*/*v*) TFA was added, and the samples were incubated for 1 h at 37 °C. Thereafter, 30 µL of 50% (*v*/*v*) acetonitrile (containing 2.5% (*v*/*v*) TFA) was added to the gel pieces and then incubated for 1 h at 30 °C. After each step, the supernatants were pooled and dried to 10 µL using a vacuum concentration system.

The digested protein spots were identified by a LC–MS/MS system (Q-TOF 6520, Agilent Technologies, Santa Clara, CA, USA), equipped with capillary pump G1382A, nano pump G2225A, autosampler G1377D, and chip cube G4240A. The LC-Chip used (Agilent Technologies) consisted of a Zorbax 300SB-C18 enrichment column (40 nL, 5 µm) and a Zorbax 300SB-C18 analytical column (75 µm × 43 mm, 5 µm). The loading flow rate was 4 µL/min and loading mobile phase was water with 0.1% formic acid. Elution from the analytical column was performed by a binary solvent mixture composed of water with 0.1% formic acid (solvent A) and acetonitrile with 0.1% formic acid (solvent B). The following gradient program was used: from 3% to 8% B in 1 min, from 8% to 40% B in 5 min, from 40% to 85% B in 1 min, and 85% B for 1 min. The chip flow rate was 300 nL/min. The MS conditions were the following: positive ion mode, Vcap of 1900 V, drying gas flow rate of 5 L/min, drying gas temperature of 350 °C, fragment voltage of 175 V, skimmer voltage of 65 V, and reference masses *m*/*z* of 149.02332 and 1221.02332. The digested samples (10 µL) were diluted in 20 µL of water with 0.1% formic acid and centrifuged for 5 min at 10,000 g, and 10 µL of the upper solution was injected. The spectra were calibrated using the mass reference standards of purine and HP-0921 (121.050873 and 922.009798, respectively; Agilent Technologies). The tandem mass spectra were retrieved using the MassHunter software (version B.04.01, Agilent Technologies). Before the MS/MS data search, a peak-list was generated by the Mascot Distiller software (version 3.2.1.0, Matrix Science, London, UK). The MS/MS data were searched against Mascot 2.4 (Matrix Science) applied to NCBInr. The search parameters were as follows: carbamidomethyl (C) and oxidation (M) were selected as fixed and variable modifications, respectively. The other parameters used were the following: taxonomy, all entries; enzyme, trypsin/P; missed cleavages, 1; peptide tolerance, ±20 ppm, and MS/MS tolerance, ±0.02 Da.

### 4.4. Bioinformatic Approach

To enrich the differentially expressed proteins with respect to specific functional terms, the protein lists were analyzed using the plug-in of the Cytoscape software: ClueGO [57] with the Gene Ontology database (release date: June 2014). The ontology selection on the base of biological processes and enrichment analysis was performed by the right-side hyper-geometric statistic test and its probability value was corrected by the Bonferroni’s method [58]. A pathway enrichment analysis of the differentially expressed proteins [59] was conducted using ClueGO software and applying database from the Kyoto Encyclopedia of Genes and Genomes (KEGG) database (release date: March 2014).

A protein interaction network of the differentially regulated proteins was analyzed using the online database resource Search Tool for the Retrieval of Interacting Genes (STRING 9.1) [60]. The protein regulation networks and protein interaction maps are in the *Susscrofa* molecular networks database. The network nodes are the proteins, and the edges represent the predicted functional associations. An edge may be drawn with up to 7 differently colored lines. These lines represent the existence of the 7 types of evidence used for predicting the associations. The interactions between the imported proteins and all proteins stored in the database were then identified.

### 4.5. Validation of Differentially Expressed Proteins by Western Blot

Western-blotting analysis was used to validate the main differentially expressed proteins. Total protein (30 µg/sample) was separated by electrophoresis (Bio-Rad, Richmond, CA, USA) on 10% SDS-PAGE, and transferred to a PVDF membrane (Millipore, Billerica, MA, USA). The blotted membrane was blocked for 2 h at room temperature in 1× TBST [0.05% Tween 20, 100 mM Tris–HCl and 150 mM NaCl (pH 7.5)] containing 5% fat-free dry milk, and then incubated under gentle agitation all the night at room temperature in the presence of the primary antibodies: HSP90B1, 1:5000 dilution of purified mouse monoclonal anti-HSPH1 antibody (Abcam, AB78902 Cambridge, UK); HSPA1A, 1:5000 dilution of purified mouse monoclonal anti-HSPA1A antibody (TDY062F, Beijing Biosynthesis Biotechnology Co., Ltd., China); Glyceraldehyde-3-phophate dehydrogenase (GAPDH), 1:2000 dilution of purified mouse monoclonal anti-GAPDH antibody (TDY062, Beijing Biosynthesis Biotechnology Co., Ltd., Beijing, China); HSPB1, 1:1000 dilution of purified rabbit polyclonal anti-HSPB1 protein antibody (Abcam, AB2790, Cambridge, UK), which were able to bind to their specific protein. The blots were extensively washed with TBST buffer for 10 min × 3 times and incubated under gentle agitation with the secondary antibodies for immunodetection. The antigenantibody interaction was carried out for 1 h, and the crossreacting proteins were detected using ECL (Perkin Elmer Life Sciences, Boston, MA, USA). The protein bands were visualized with a chemiluminescence substrate using a gel-imaging system (Tanon Science and Technology, Shanghai, China) with Image Analysis Software (National Institutes of Health, Bethesda, MD, USA). In all instances, density values of bands were corrected by subtraction of the background values. GAPDH was used as the internal reference protein. Bands were standardized to the density of GAPDH and normalized fold expression represented as a ratio of each protein to GAPDH.

### 4.6. Determination of Anti-oxidant Enzyme Activities in Finishing Pig Liver

Anti-oxidant enzyme activities of glutathione peroxidase (GPX) and glutathione-*S*-transferase (GST) and the content of the reduced glutathione (GSH) and oxidized glutathione (GSSG) in liver were measured using the corresponding assay kits (Nanjing Jiancheng Bioengineering Institute, Nanjing, China) according to the manufacturer’s instructions. Results are expressed on the basis of tissue protein content.

### 4.7. Statistical Analysis

Statistical analyses were performed with one-way ANOVA using SAS software (version 8.1; SAS Institute, Cary, NC, USA). All data were expressed as mean ± standard deviation (SD). A Duncan’s multiple range test was used to compare the difference among groups, and a difference at *p* < 0.05 was taken to indicate statistical significance.

## 5. Conclusions

To our knowledge, our study provides novel information about the effects of chronic HS on the liver of finishing pigs at the proteomic level. Our results indicate that chronic HS alters the expression of hepatic proteins, which are related to the regulation of oxidative stress, redox state, and apoptosis. Furthermore, we showed that HS triggers the immune response independent of food restriction. Chronic HS induced hepatic cellular apoptosis, while food restriction also induced stress and cellular apoptosis. To neutralize the effects of chronic HS, we suggest that the liver responds by up-regulating HSPs to maintain protein folding structures, which enhances the anti-oxidant pathway for a balanced redox state, and leads to the overexpression of the proteasome for the degradation of oxidized or misfolded proteins. In conclusion, our results contribute novel insights into the molecular mechanisms of chronic HS in the pig liver.

## Figures and Tables

**Table 1:** Effects of *ad-libitum* feed intake in thermal neutral conditions (TN; 22 °C), *ad-libitum* feed intake in chronic heat stress conditions (HS; 30 °C), or pair-feeding in thermal neutral conditions (PF) on liver ALW and RLW.

Parameter	TN	HS	PF	*p* Value
ALW (kg)	1.65 ± 0.10 ^a^	1.48 ± 0.11 ^b^	1.44 ± 0.05 ^b^	0.009
RLW (%)	2.01 ± 0.13 ^a^	1.75 ± 0.23 ^b^	1.84 ± 0.22 ^b^	0.04

Data are mean ± SD, *n* = 8 pigs for each group. Values with different letters (^a^ and ^b^) are significantly different (*p* < 0.05). ALW = Absolute liver weight; RLW = absolute liver weight (kg) corrected for body weight (kg).

**Table 2:** Biochemical information about proteins differentially expressed in the liver of finishing pigs exposed to chronic heat stress or reduced feed intake.

Spot No. ^a^	Accession No. ^b^	Protein Name	Short Name	Score ^c^	PM ^d^	Sequence Coverage (%)	Theoretical Mr(kDa)/pI ^e^	Functions
**Stress Response and Immune Defense (9)**								
**7**	gi|545829329	PREDICTED: haptoglobin isoform X1	HP	176.71	15	41.53	45.21/6.13	Acute-phase response; immune defense
**11**	gi|47523016	Endoplasmin precursor	HSP90B1	119.66	6	7.59	92.47/4.76	Innate immune response; response to ER stress
**20**	gi|345441750	Heat shock 70 kDa protein 8	HSPA8	166.46	12	21.67	70.90/5.37	Chaperone; stress response
**21**	gi|350579657	PREDICTED: 78 kDa glucose-regulated protein	HSPA5	141.01	13	19.27	72.33/5.07	Chaperone; stress response
**28**	gi|47523270	α-1-Antichymotrypsin 2 precursor	SERPINA3	101.78	17	57.32	47.65/5.33	Inflammatory response; acute-phase response
**33**	gi|350582856	PREDICTED: elongation factor 1-delta isoformX2	EEF1D	102.57	8	29.69	31.12/4.90	Transcription regulation; heat shock protein binding
**35**	gi|47522774	Heat shock protein HSP 90-α	HSP90AA1	170.82	10	13.11	84.66/4.94	Innate immune response; stress response
**37**	gi|55668280	Hsp27	HSPB1	204.53	14	54.11	22.78/5.98	Chaperone; stress response
**40**	gi|39777368	Heat shock protein 70.2	HSPA1A	160.30	17	20.75	70.05/5.47	Chaperone; stress response
**Antioxidant System (10)**								
**4**	gi|118403788	Glutathione S-transferase mu 2	GSTM2	198.75	14	42.66	25.74/5.99	Glutathione metabolic; cellular detoxification
**16**	gi|67038668	DJ-1 protein	PARK7	228.29	16	57.68	19.89/6.32	Inflammatory response; oxidative stress response
**53**	gi|545815980	PREDICTED: sulfotransferase 1C4-like (EC 2.8.2)	SULT1C4	154.30	10	30.65	35.52/8.22	Transferase
**59**	gi|345199274	Glutaredoxin 3	GLRX3	79.56	7	20.36	37.43/5.31	Cell redox homeostasis
**47**	gi|194036227	PREDICTED: selenium-binding protein 1	SELENBP1	339.53	34	65.68	52.39/5.93	Selenium binding
**10**	gi|347582636	Aldehyde dehydrogenase 1 family, member L1	ALDH1L1	154.87	13	16.31	98.83/5.63	Cellular aldehyde metabolic process
**19**	gi|545822151	4-trimethylaminobutyraldehyde dehydrogenase	ALDH9A1	132.85	5	9.15	53.80/5.69	Cellular aldehyde metabolic process
**30**	gi|160419232	Proteasome subunit β type-7	PSMB7	136.91	7	25.60	27.89/8.60	Protease; hydrolase activity
**32**	gi|346421411	Proteasome 26S subunit ATPase 3	PSMC3	146.53	15	31.36	49.20/5.13	Protease; hydrolase activity
**65**	gi|346421372	Ferritin, light polypeptide	FTL	107.45	6	41.08	21.30/5.70	Cellular iron ion homeostasis
**Cellular Proliferation and Apoptosis (11)**								
**9**	gi|304365428	Protein disulfide-isomerase A3 precursor	PDIA3	120.17	7	24.27	56.78/5.98	Cell redox homeostasis; apoptosis
**13**	gi|358009193	Prolyl 4-hydroxylase β polypeptide	P4HB	235.16	6	14.76	57.12/4.76	Cell redox homeostasis; apoptosis
**12**	gi|46397561	Fas-associating death domain-containing protein	FADD	174.58	12	38.39	23.28/5.48	Innate immunity; apoptosis
**18**	gi|323145752	Interferon-stimulated protein 60	IRF9	78.77	4	9.43	43.70/5.58	Signal transduction; apoptosis
**25**	gi|408360214	RecName: Full = Vimentin	VIM	202.05	29	50.64	53.65/5.05	Programmed cell death
**27**	gi|311258550	PREDICTED: EF-hand domain-containing protein D2	EFHD2	184.90	17	54.59	26.70/5.15	Apoptosis
**31**	gi|47716872	Galectin-1, partial	LGALS1	100.62	5	41.04	14.72/5.30	Regulation of apoptotic process
**46**	gi|115499496	NDRG2	NDRG2	110.55	3	8.69	40.80/5.08	Cell differentiation
**54**	gi|335308924	PREDICTED: γ-glutamylcyclotransferase-like isoformX1 (EC 2.3.2.4)	GGCT	230.41	15	59.04	21.01/5.07	Apoptosis; glutathione homeostasis
**70**	gi|335284397	PREDICTED: major vault protein isoform 1	MVP	190.97	30	33.67	99.33/5.34	Apoptosis
**64**	gi|350581487	PREDICTED: 14-3-3 protein γ isoform X1	YWHAG	255.37	20	65.31	28.30/4.80	Programmed cell death
**Metabolism (10)**								
**24**	gi|298104076	Enoyl-CoA hydratase, mitochondrial	ECHS1	139.16	11	31.38	31.39/8.34	Fatty acid metabolism
**52**	gi|311271975	PREDICTED: short/branched chain specific acyl-CoA Dehydrogenase, mitochondrial	ACADSB	187.41	14	32.71	47.49/6.53	Fatty acid metabolism
**50**	gi|349732238	Transaldolase	TALDO1	119.81	18	39.17	37.54/6.36	Carbohydrate metabolism
**38**	gi|335298275	Glyoxalase domain-containing protein 4	GLOD4	236.08	12	45.45	34.79/5.40	Carbohydrate metabolism
**63**	gi|2642486	Cytochrome b5	CYB5A	252.85	17	69.15	15.33/4.86	vitamin metabolic process
**61**	gi|1931	Carboxylesterase precursor	CES1	298.47	36	48.27	62.52/6.15	lipid catabolic process
**15**	gi|33323483	Cellular retinol binding protein 1	RBP1	152.93	7	38.52	15.85/4.99	vitamin A metabolic process
**51**	gi|343780946	D-dopachrome decarboxylase	DDT	141.84	10	59.32	12.71/6.72	melanin biosynthetic process
**67**	gi|349732258	Uroporphyrinogen decarboxylase	UROD	209.27	13	32.43	40.79/5.77	Porphyrin biosynthesis
**48**	gi|335294970	PREDICTED: tetratricopeptide repeat protein 36	TTC36	86.78	5	12.84	20.90/5.02	Amino acid metabllism
**Signaltransduction (2)**								
**41**	gi|346644814	Sorcin isoform 2	SRI	111.12	7	11.65	21.68/5.32	Calcium homeostasis
**68**	gi|116175265	Regucalcin	RGN	119.57	7	35.07	33.25/5.89	Calcium homeostasis
**Cytoskeleton (3)**								
**17**	gi|195562237	Actin related protein 2/3 complex subunit 5	ARPC5	137.21	7	38.41	16.32/5.47	Cell migration
**34**	gi|545825420	PREDICTED: keratin, type I cytoskeletal 18, partial	KRT18	102.03	15	32.24	48.06/5.34	Cell cycle
**43**	gi|297307133	Protein canopy homolog 2 precursor	CNPY2	201.68	9	46.15	20.65/4.81	Cytoskeletal structure

^a^ Spot number as given in Figure 2; ^b^ Accession number according to the NCBI database; ^c^ Protein score is −10 × log (*p*) where *p* is the probability that the observed match is a random event. Protein scores of 59 or greater indicate a significant match (*p* < 0.05) with the named protein; ^d^ Number of query matched peptides; ^e^ Theoretical Mr (kDa)/pI: molecular mass/isoelectric point of the predicted protein.

**Table 3:** Enriched KEGG pathway-based sets of differentially expressed proteins in the liver of finishing pigs subjected to chronic heat stress or reduced feed intake ^a^.

Pathway Name	Count	Protein	*p* Value	*q* Value
Ascorbate and aldarate metabolism	2	ALDH9A1, RGN	8.45 × 10^−3^	4.94 × 10^−2^
Fatty acid degradation	3	ACADSB, ALDH9A1, ECHS1	1.47 × 10^−3^	1.47 × 10^−2^
Proteasome	2	PSMB7, PSMC3	7.17 × 10^−3^	7.89 × 10^−2^
Unfolded protein response	6	HSPA5,HSPA8, P4HB, HSPA1A, PDIA3 HSP90B1, HSP90AA1	1.91 × 10^−5^	2.48 × 10^−4^
Antigen processing and presentation	5	HSP90AA1, HSPA1A, HSPA5, HSPA8, PDIA3	5.78 × 10^−5^	6.93 × 10^−4^

^a^ The number of count refers to the amount of proteins involved in the extended KEGG network and pathway; *p* values are calculated according to a hypergeometric test; *q* values represent p-values corrected for multiple testing using the false discovery rate method. For an explanation of abbreviations, please refer to Table 2.

**Table 4:** Composition of the experimental diet.

Item	Content
Ingredient	g/kg
Corn	662.0
Soybean meal, 42.8% CP	200.0
Wheat bran	65.0
Wheat middlings	40.0
Limestone	10.0
Dicalcium phosphate	6.0
Salt	4.0
Premix ^1^	10.0
l-Lysine·HCl	3.0
Chemical composition ^2^	g/kg
Digestive energy (MJ/kg)	13.39
Crude protein	157.3
Calcium	6.5
Total phosphorus	4.1
Available phosphorus	1.7
Lysine	9.2
Met + Cys	5.4

^1^ Premix provided the following per kg of complete diet for finisher pigs: vitamin A, 2512 IU; vitamin D_3_, 1200 IU; vitamin E, 34 IU; vitamin K_3_, 1.5 mg; vitamin B_12_, 17.6 µg; riboflavin, 2.5 mg; pantothenic acid, 6.8 mg; niacin, 20.3 mg; choline chloride, 351 mg; Mn, 10 mg; Fe, 50 mg; Zn, 50 mg; Cu, 10 mg; I, 0.3 mg; Se, 0.3 mg. ^2^ Calculated values.
